# Efficient Copper Biosorption by *Rossellomorea* sp. ZC255: Strain Characterization, Kinetic–Equilibrium Analysis, and Genomic Perspectives

**DOI:** 10.3390/microorganisms13081839

**Published:** 2025-08-07

**Authors:** Hao-Tong Han, Han-Sheng Zhu, Jin-Tao Zhang, Xin-Yun Tan, Yan-Xin Wu, Chang Liu, Xin-Yu Liu, Meng-Qi Ye

**Affiliations:** 1 Marine College, Shandong University, Weihai 264209, China; 13555222125@163.com (H.-T.H.);; 2 Shenzhen Research Institute, Shandong University, Shenzhen 518057, China; 3 Weihai Research Institute of Industrial Technology of Shandong University, Weihai 264209, China

**Keywords:** bioremediation, heavy metal pollution, copper removal, genomics

## Abstract

Heavy metal pollution, particularly copper contamination, threatens the ecological environment and human survival. In response to this pressing environmental issue, the development of innovative remediation strategies has become imperative. Bioremediation technology is characterized by remarkable advantages, including its ecological friendliness, cost-effectiveness, and operational efficiency. In our previous research, *Rossellomorea* sp. ZC255 demonstrated substantial potential for environmental bioremediation applications. This study investigated the removal characteristics and underlying mechanism of strain ZC255 and revealed that the maximum removal capacity was 253.4 mg/g biomass under the optimal conditions (pH 7.0, 28 °C, and 2% inoculum). The assessment of the biosorption process followed pseudo-second-order kinetics, while the adsorption isotherm may fit well with both the Langmuir and Freundlich models. Cell surface alterations on the Cu(II)-treated biomass were observed through scanning electron microscopy (SEM). Cu(II) binding functional groups were determined via Fourier transform infrared spectroscopy (FTIR) analysis. Simultaneously, the genomic analysis of strain ZC255 identified multiple genes potentially involved in heavy metal resistance, transport, and metabolic processes. These studies highlight the significance of strain ZC255 in the context of environmental heavy metal bioremediation research and provide a basis for using strain ZC255 as a copper removal biosorbent.

## 1. Introduction

With the continuous advancement of industrialization, heavy metal contamination is recognized as the most critical environmental and human health challenge globally [1,2]. Cu, Cr, and Ni have been identified as the most serious contaminants according to the US EPA Sediment Quality Guidelines (SQGs) [3]. As persistent contaminants, heavy metals accumulate in the environment and contaminate food chains [4]. The accumulation of potentially toxic heavy metals in living organisms has significant health risks for their consumers, including humans [5]. The negative effects of heavy metals on plants include inhibiting photosynthesis, reducing seed germination, and decreasing enzyme activity and chlorophyll synthesis [6]. Furthermore, heavy metals exert profound adverse effects on human health, including various types of organ damage and diseases, anemia, skin irritation, impaired muscle function, short-term memory loss, mental retardation, etc., even at low concentrations [7].

As is well known, copper is essential for numerous biochemical processes [8]. It is necessary for the functioning of many enzymes in living organisms [9]. However, exposure to copper above acceptable limits would harm human health and the environment [10]. For instance, excessive Cu(II) levels in the human body can lead to anemia, respiratory infections, and liver and kidney failures [11]. This dual nature of copper—as both a vital nutrient and a potential toxin—underscores the importance of maintaining its concentration within optimal ranges in biological and environmental systems.

Given the extreme severity of heavy metal pollution, the development of efficient methods for removing heavy metal pollutants has become a major research focus in recent years [12]. Conventional wastewater treatment approaches, including electrocoagulation, ultrafiltration, ion-exchange processes, and osmosis separation systems, demonstrate notable drawbacks such as inconsistent removal efficiency, excessive chemical consumption, high cost, and secondary pollution from residual sludge [13]. In contrast, biosorption has emerged as an eco-friendly alternative with operational advantages in terms of cost-effectiveness and sustainability. Extensive research has been conducted on biosorbents derived from various biological substrates, including microbial biomass, macroalgal species, industrial byproducts, agricultural residues, natural plant-derived materials, and other biopolymers [14]. Among these various biosorbents, bacteria are better suited to bioremediation due to their superior surface area-to-volume ratios, rapid metal adsorption rates, natural abundance, and economic viability [15]. Biosorption encompasses multiple complex mechanisms such as extracellular and intracellular metal complexation, precipitation, and methylation [16]. The components and various proteins in the cell wall play crucial roles in metallic ion immobilization through diverse interactions [17]. Bacteria also have a large number of functional groups such as -COOH, -SH, and -OH on the microbial surfaces, which actively participate in metal adsorption through various coordination chemistries [18].

In our previous research, we found that *Rossellomorea* sp. ZC255 is promising for the bioremediation of Cu(II), and the minimal inhibitory concentration (MIC) of Cu(II) required for the growth of strain ZC255 is 1600 mg/L [19]. In this study, we further evaluated the external parameters, such as the pH, temperature, and inoculation amount, that impact the removal process. The characterization of the biosorption behavior of biomass is determined via kinetics and equilibrium. The bioaccumulation of copper in bacterial cells was also investigated using electron microscopy and spectroscopic analyses. In addition, genome sequences were used to annotate functional genes associated with heavy metal resistance and copper adsorption.

## 2. Materials and Methods

### 2.1. Batch Removal Experiments

The effects of pH, temperature, and inoculation amount on the removal capacity of strain ZC255 were systematically studied. Batch removal experiments were conducted in 150 mL samples of Marine broth 2216 (MB; Becton-Dickinson, Franklin Lakes, NJ, USA) containing 40 mg/L of Cu(II). Temperature effects were evaluated under a controlled rotary shaker (ZWY-200D, Zhicheng Analytical Instrument Manufacturing Co., Ltd., Shanghai, China) (120 rpm; 4-day incubation) across five thermal conditions: 15 °C, 20 °C, 28 °C, 37 °C, and 40 °C. To determine the optimum pH for Cu(II) removal, the pH value was adjusted to 5.5, 6.0, 6.5, 7.0, and 7.5 by using 0.1 M HCl and 0.1 M NaOH, followed by stabilization with using 20 mM buffer systems: MES (pH 5.5 and 6.0), PIPES (pH 6.5 and 7.0), and HEPES (pH 7.5). Different inoculation amounts (1%, 2%, 3%, 4%, and 5%) were added to 150 mL of MB containing Cu(II) to investigate the optimum inoculation amount.

The liquid phase was collected via centrifugal separation at 8000 rpm for 5 min. The residual Cu(II) concentration in the supernatants was measured using an atomic absorption spectrophotometer (Model: 900H, Perkin Elmer, Waltham, MA, USA) [20]. Before measurement, the supernatants were diluted with deionized water. The absolute removal capacity (Q_e_) of Cu(II) was calculated using the following established formula [9]:

(1) $$Q_{e}=(C_{0}-C_{t})\times V/M$$

where *C_0_* and *C_t_* represent the initial and equilibrium Cu(II) concentration (mg/L), *V* is the solution volume (mL), *M* is the bacterial biomass (g), and *Q_e_* is the absolute removal capacity of strain ZC255 (mg/g). All experimental determinations followed a standardized triplicate protocol with randomized sample processing sequences.

### 2.2. Kinetic Models for Batch Experiments

A crucial factor in assessing the viability and efficiency of the biosorption process is the kinetic mechanism of biosorption [21]. The batch experiment was performed for 16 h, 32 h, 48 h, 64 h, 80 h, 96 h, and 112 h, with a 160 mg/L initial Cu(II) concentration. The experimental data were utilized to facilitate an exacting evaluation of the biosorbent’s adsorption rate and capacity, employing first-order and second-order kinetic models as the analytical frameworks.

The Lagergren pseudo-first-order adsorption kinetics equation is as follows [22]:

(2) $$log\left(q_{e}-q_{t}\right)=logq_{e}-k_{1}\times t/2.303$$

The Lagergren pseudo-second-order adsorption kinetics equation is as follows [23]:

(3) $$t/q_{t}=1/k_{2}\times q_{e}^{2}+t/q_{e}$$

where *q_e_* and *q_t_* are the biosorption quantity at equilibrium (mg/g) and at time (h), *t* is the reaction duration (h), and *k_1_* and *k_2_* are constants.

### 2.3. Equilibrium Isotherm Models for Batch Assays

The adsorption isotherm model was employed to analyze and differentiate the biosorption characteristics of various biosorbents [24]. Equilibrium isotherm studies were conducted under conditions of a certain pH, an inoculation amount of 2%, contact time of 112 h, and temperature of 28 °C, with different Cu(II) concentrations. The remaining experimental methods were consistent with those described previously. The obtained data were fitted to the Langmuir and Freundlich isotherm models.

For the Langmuir isotherm model, the following equation was used [25]:

(4) $$q_{e}=K_{L}q_{max}C_{e}/\left(1+K_{L}C_{e}\right)\;$$

For the Freundlich isotherm model, the following equation was used [26]:

(5) $$q_{e}=K_{F}\times C_{e}^{1/n}$$

where *q_e_* is the quantity in milligrams of metal adsorbed per unit of biomass (mg/g); *K_L_*, *K_F_*, and n are constants; *q_max_* is the theoretical monolayer saturation capacity (mg/g); and *C_e_* is the equilibrium metal concentration (mg/L).

### 2.4. SEM Analysis

To elucidate copper ion-induced cytomorphological changes, scanning electron microscopy (model Nova NanoSEM450, FEI, Portland, OR, USA) was adopted in this study. The strains were incubated in Cu(II)-containing and Cu(II)-free media, respectively. After incubation for 48 h, cellular pellets were obtained via centrifuging at 8000 rpm for 5 min, which were then washed with 0.1 M phosphate buffer solution (PBS: pH 7.0), and fixed in 2.5% glutaraldehyde solution for at least 4 h. Subsequently, dehydration involved sequential gradients (50%, 70%, 80%, 90%, and 100% ethanol) with 10 min immersion per concentration tier. The obtained samples were gold-plated before analysis with a stable voltage of 10 keV.

### 2.5. FTIR Analysis

Surface functional group dynamics during metal biosorption were investigated through comparative Fourier transform infrared spectroscopy. After centrifuging (8000 rpm) the control and Cu(II)-treated organisms separately, the bacterial precipitate was frozen at −80 °C for 24 h, followed by lyophilization with a freeze-dryer, and then the samples were pressed in KBr pellets according to a ratio of 1:50, making them sufficiently homogenized and finely ground. After that, the mixture was pressed into tablets and placed in the spectrophotometer (Thermo Nicolet iS5, Waltham, MA, USA) within the range of 4000–500 cm^−1^ [27].

### 2.6. Genome Annotation and Analysis of ZC255

The genomic DNA of strain ZC255 was isolated utilizing a genomic DNA isolation kit (Takara Biomedical Technology Co., Ltd., Beijing, China). Whole genome sequencing was performed through the Illumina sequencing platform (MajorBio Co., Shanghai, China). In brief, DNA was fragmented to ~400 bp via Covaris M220 acoustic shearing, followed by library construction using NEXTFLEX Rapid DNA-Seq Kit (Bioo Scientific, Austin, TX, USA). The workflow involved sequential DNA end-repair and phosphorylation, 3′ A-tailing, adapter ligation, and PCR-based library amplification. The sequencing libraries underwent paired-end Illumina sequencing (2 × 150 bp) using the Illumina Novaseq 6000 (Illumina Inc., San Diego, CA, USA) tool. Then, sequencing-derived raw reads underwent quality filtration via fastp (version 0.19.6) [28] prior to genome reconstruction using SOPA de novo v2.04. The quality of the genomes was assessed through CheckM v1.1.6 [29]. The genome sequences were annotated using the RAST server. The predicted CDSs were annotated using sequence alignment tools from the Clusters of Orthologous Groups (COG), Gene Ontology (GO), and Kyoto Encyclopedia of Genes and Genomes (KEGG) databases. GO is an extensively employed bioinformatics concept that unifies the genes and gene products of all species [30]. BlastKOALA v3.1 was used to scan the KEGG database and examine the metabolic pathways [31]. The genome-wide comparisons and annotations of COG and GO were performed using EggNOG-mapper v2.1 [32].

## 3. Results and Discussion

### 3.1. Batch Biosorption Experiment

#### 3.1.1. Effect of Solution pH

**Figure 1:**
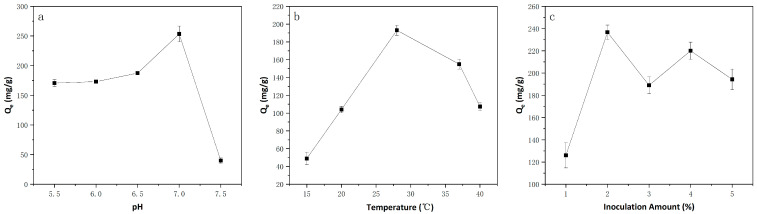
Effects of different parameters, (**a**) pH, (**b**) temperature, and (**c**) inoculation amount, on removal capacity of Cu(II) onto strain ZC255.

As is widely recognized, the form of the compounds in water is significantly influenced by the solution pH [33]. The solution acidity fundamentally governs the competitive interactions between dissolved metal cations and biomass-associated reactive sites [34]. The influence of the pH on the effectiveness of Cu(II) removal is shown in Figure 1. The removal capacity exhibited a direct proportional relationship to pH elevation, achieving optimal performance at pH 7.0. At higher pH values, the removal capacity decreased significantly. The reason for such a situation might be that cell wall ligands exhibit a strong affinity for hydronium ions at lower pH values. Repulsive forces, acting on these ligands, limit the approach of metal ions [35]. The negative charges on the cell surface increase when the pH value increases because more ligands, including imidazole, carboxyl, phosphate, and amino groups, are exposed. This phenomenon subsequently leads to the attraction of positively charged metal ions and subsequent biosorption on the cell surface [10].

#### 3.1.2. Effect of Temperature

As demonstrated by EI-Gendy and EI-Bondkly et al. [36], temperature plays a pivotal role in heavy metal removal by live biosorbents, as it directly influences biomass growth and metabolic activity. A decline in removal effectiveness may result from departures from the optimal temperature range. As shown in Figure 1, the removal capacity of strain ZC255 for Cu(II) increased as the temperature increased from 15 °C to 28 °C but declined at higher temperatures. According to Kayalvizhi et al. [37], it has been established that an increase in temperature activates additional binding sites on the biomass surface and widens the pores within the biosorbent material. Concurrently, a higher temperature can enhance the mass transfer coefficient due to the acceleration of the collisions between heavy metal ions and active groups located on the biosorbent surface [38], thereby accelerating the reaction rate [4]. At the same time, the reduced removal capacity observed at low temperatures is likely attributable to the inactivation of various enzyme activities [39] and a decrease in membrane fluidity [40]. When the temperature exceeds the optimal range, the growth of the biomass may be inhibited, resulting in a reduced removal capacity.

#### 3.1.3. Effect of Inoculation Amount

The inoculation amount has been demonstrated to influence the quantity of the biosorbent surface area and active binding site availability [41]. According to the results shown in Figure 1, a 2% inoculation quantity was sufficient to obtain the maximal removal capability. As the inoculation amount increased, the adsorption surface area increased, leading to an initial increase in the removal capacity [42]. However, excessive inoculation concentrations reduce metal removal efficiency. One of the main causes of these phenomena is the agglomeration of biomass at increasing concentrations, which lowers the effective surface area accessible for removal processes [43]. Similar findings were also made by Selatina [44] and Lu et al. [45].

In summary, strain ZC255 exhibited the highest Cu(II) removal capacity, which was 253.4 mg/g of biomass under the conditions of 28 °C, a pH value of 7.0, and an inoculation amount of 2%. The initial and remaining concentrations of Cu(II) in the solution were 79.2 and 57.5 mg/L. The optimized removal rate was increased by 10.7% compared to the previous experimental results [19]. These optimal conditions are within a common range rather than extreme values, making them applicable to various environments. Additionally, considering cost issues, biological adsorption for removing heavy metal pollutants is rarely conducted at high temperatures, and researchers hope to carry out this process at room temperature [38]. The optimal temperature of 28 °C can better reduce costs.

### 3.2. Kinetic Models for Batch Experiment

**Figure 2:**
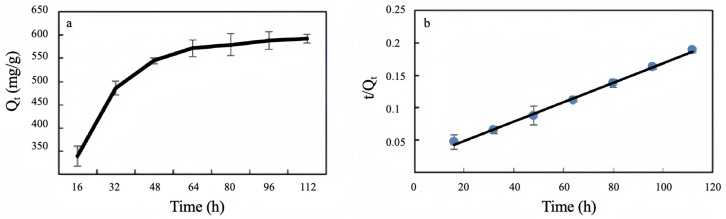
The (**a**) first-order and (**b**) second-order kinetics during Cu(II) biosorption by strain ZC255.

Adsorption kinetics refers to the relationship between the rate of biosorption and the duration of the biosorption process [46]. The biosorption kinetics of Cu(II) by strain ZC255 are shown in Figure 2. The Cu(II) removal capacity of strain ZC255 revealed a time-dependent trend, increasing progressively with increasing contact time. When the contact time reached 80 h, the removal capacity increased slowly, approaching an equilibrium state. This behavior can be attributed to the two phases of the removal process. The initial phase is characterized by rapid surface binding, followed by a subsequent phase characterized by slow intracellular diffusion [42].

The second-order adsorption kinetics model better describes the adsorption behavior of strain ZC255, as indicated in Table S1, as the first-order adsorption kinetics correlation coefficient R^2^ (0.915) of strain ZC255 is smaller than the second-order adsorption kinetics correlation coefficient R^2^ (0.997). The second-order kinetic model is based on the premise that the rate of sorption is governed by chemical sorption [45]. Consequently, biosorption by the ZC255 strain is predominantly governed by the chemical interactions that occur between Cu(II) and the surface sites of the bacterial biomass [47].

### 3.3. Equilibrium Isotherm Models for Batch Assays

**Figure 3:**
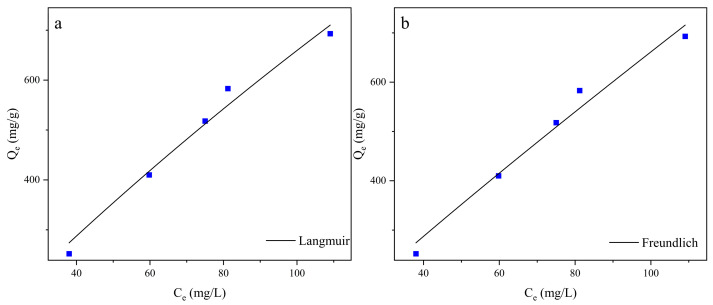
Cu(II) biosorption by strain ZC255: the (**a**) Langmuir and (**b**) Freundlich models.

The isotherm is indicative of the equilibrium relationship between the biosorbent’s metal adsorption and the residual metal concentration in the solution, thereby demonstrating the biosorption capacity of the biosorbent [48]. The fitting results are shown in Table S2 and Figure 3. The Langmuir isotherm assumes a uniform surface characterized by single-layer adsorption, monolayer surface coverage, and homogeneously available adsorption sites [49]. This model is frequently employed to predict the biosorption of solutes onto the biosorbent, where the characteristics of binding sites exhibit considerable variability [21]. Given the varying affinity for solutes at various surface locations [4], the Freundlich isotherm characterizes adsorption on a heterogeneous surface, including several adsorption layers and non-ideal sorption [50]. Biosorption energy and behavior can be predicted under various conditions, an important tool for wastewater treatment and biosorption applications [51]. Given that the R^2^ values of the two models are very close, both models clearly describe the adsorption process of strain ZC255 well. In other researchers’ experiments, we have found that a similar situation exists (Huang and Liu [52]; Joo et al. [53]; Lu et al. [45]), but no one has provided an explanation for how these two different models can match the experimental data simultaneously [54].

### 3.4. SEM Analysis

**Figure 4:**
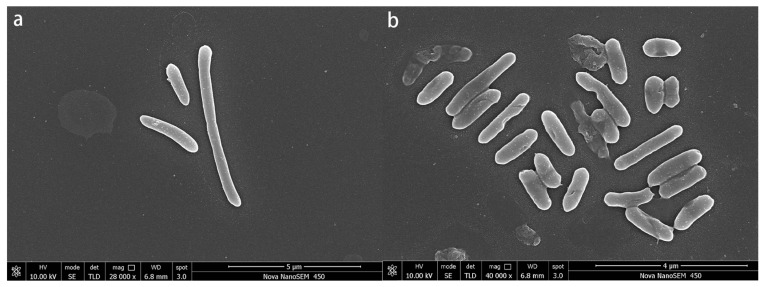
A scanning electron micrograph of strain ZC255 under the control condition (**a**) and the Cu(II) stress condition (**b**).

The SEM images indicate distinctions between strain ZC255 under the control condition and the ones subjected to Cu(II) stress (Figure 4). Cells treated with Cu(II) revealed considerable morphological alterations. The cells under control conditions exhibited a rod-shaped morphology with an intact and smooth surface. In contrast, under the Cu(II)-stressed conditions, the cells appeared denser and distorted and adhered to one another. Moreover, the specimens exhibited irregularities, fissuring, and the formation of wrinkles on the surface, attributable to the deleterious effects of Cu(II). It suggests that the extent of bacterial cell adhesion and physical disintegration is indicative of a reduction in the total surface area exposed to heavy metal toxicity [55]. In addition, following the adsorption of Cu(II), a substantial number of flocs were observed on the surface of the strain. The redox process, which produces alkaline compounds, might cause this phenomenon [56]. These alterations imply that the Cu(II) leads to cell surface deformation and injury during Cu absorption.

### 3.5. FTIR Analysis

**Figure 5:**
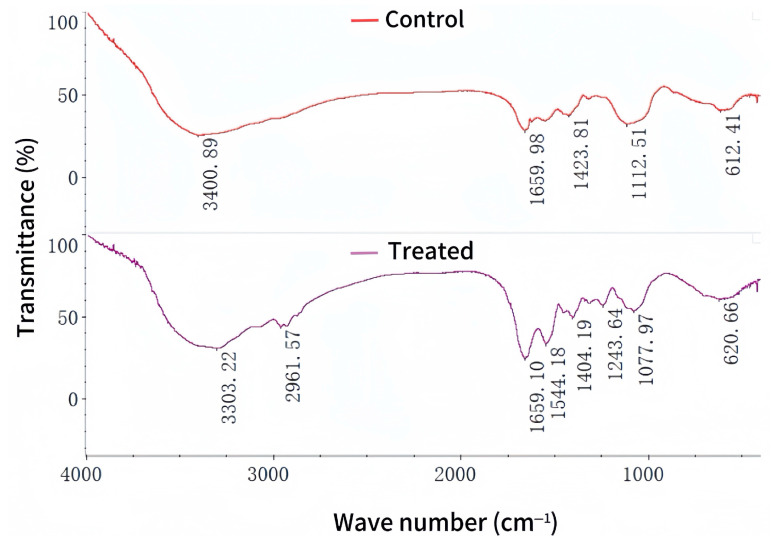
Fourier transform infrared spectroscopy spectra of control and Cu(II)-treated cells.

To study the interaction between Cu(II) and the functional groups of the bacterial cell wall of strain ZC255, the strain was analyzed before and after the adsorption of Cu(II). All the observed bands and their assignments are shown in Table S3. The ion-exchange process on the cell surface leads to the biosorption of metal ions. Several functional groups, such as amino, phosphate, carboxylate, and hydroxyl groups, can bind with metal ions [57]. The characteristic peaks were identified through previous studies, which provided references for interpreting the FTIR spectra [21,55,58,59,60]. In the FTIR spectral analysis of strain ZC255 (Figure 5), we observed peak shifting from 3400 to 3303 cm^−1^, representing the bonded -OH stretching vibration, the -NH stretching of the protein, and the acetamido group of the chitin fraction. In the region between 3000 and 2800 cm^−1^, the C-H stretching vibrations of -CH_3_ are visible. The -CN bending vibration from 1112 to 1077 cm^−1^ and peak shifting from 612 to 620 cm^−1^ indicated the binding of Cu(II) to -NH_2_ and -NO_2_. In addition, certain novel peaks of N-H bending vibration or -CN stretching vibration at 1544 cm^−1^ and -COOH stretching vibration at 1243 cm^−1^ were observed in Cu(II)-treated bacterial cells. The appearance of and change in the peaks of Cu(II)-treated bacterial cells illustrate the activity of different function groups, which directly influence biosorption.

### 3.6. Genome Annotation and Analysis of ZC255

Strain ZC255 has a genome size of 4,775,586 bp, a G + C content of 42.5 mol%, 4275 genes, 11 rRNAs, and 107 tRNAs. The sequence data are publicly available in the NCBI database (accession number PRJNA1099410).

**Figure 6:**
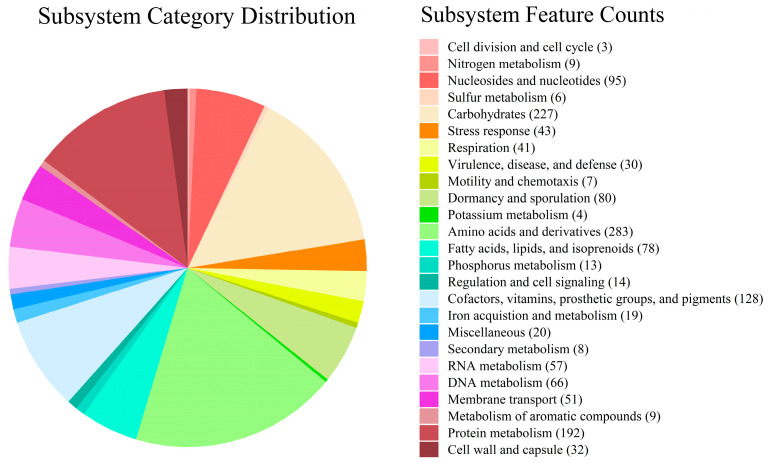
The subsystem distribution of strain ZC255 based on the RAST annotation server.

A total of 296 subsystems were annotated in RAST, covering 1515 genes (23%) (Figure 6). Regarding membrane transport (51), protein secretion system, type II (2); uni-, sym-, and antiporters (10); cation transporters (6); protein translocation across the cytoplasmic membrane (7); TRAP transporters (5); protein and nucleoprotein secretion system, type IV (9); and membrane transport, no subcategories (12) were identified. Notably, we found a copper transport system (5) in cation transporters, further demonstrating the ability of copper removal by strain ZC255.

**Figure 7:**
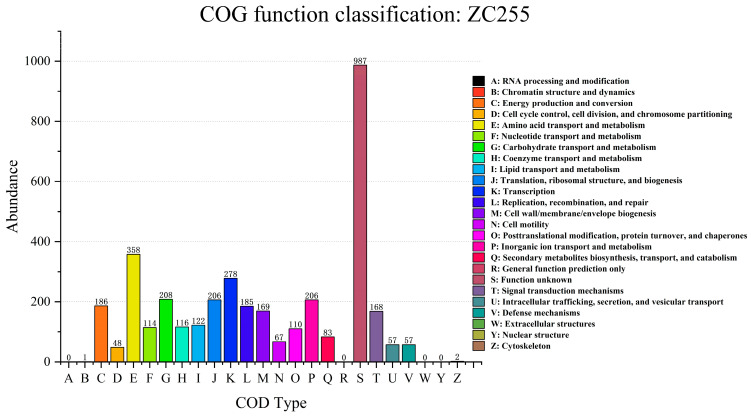
Clusters of Orthologous Genes classification statistics of strain ZC255 based on the eggNOG database.

A comparison was made using the Evolutionary Genealogy of Genes: Non-supervised Orthologous Groups (eggNOG) database. Then, the genome of strain ZC255 was subjected to COG functional annotation analysis (Figure 7). Next, 3728 genes were annotated based on the COG database. We found several genes associated with Cu(II) homeostasis (copA, copB, copZ, cutC, csoR, and dnaK). copA and copB encode two integral membrane P-type ATPases, essential for transporting Cu(II) into cells under conditions where Cu(II) is limited [61]. The expression of the cop operon is controlled by the transcriptional repressor csoR and the chaperone proteins copZ [62]. cutC has been described as “Participates in the control of copper homeostasis”. dnaK is also a gene associated with copper detoxification [63]. In addition, our research identified the presence of genes related to resistance or detoxification in response to a variety of metals, such as Mn (mntP, mntH-2, mntB, mntC, mntD, mntR, and ytaF), Zn (cat, ymfH, rasP, zupT, ypeQ, and yhaK), Cr (chr), Pt (cadA and czcD), and As (arsB-2 and arsC). At the same time, we identified a number of antioxidant genes (sadA, sodF, sodC, katE, katG, and htpX). The presence of these genes proves that strain ZC255 can not only bioaccumulate Cu(II) but also has resistance to other heavy metals, indicating that the strain can grow unaffected even under heavily polluted wastewater conditions.

Additionally, we identified a number of genes encoding antibiotic resistance (Table S4). Strain ZC255 has been found to exhibit heavy metal–antibiotic dual resistance in our previous study [19]. It can be hypothesized that strain ZC255 is co-selective, like other strains with heavy metal–antibiotic dual resistance [64], due to the tight arrangement of genes, which may be subject to combined transmission in cases of horizontal gene transfer [65]. The presence of these genes further confirms our conclusions, indicating that the strain is extremely resistant.

**Figure 8:**
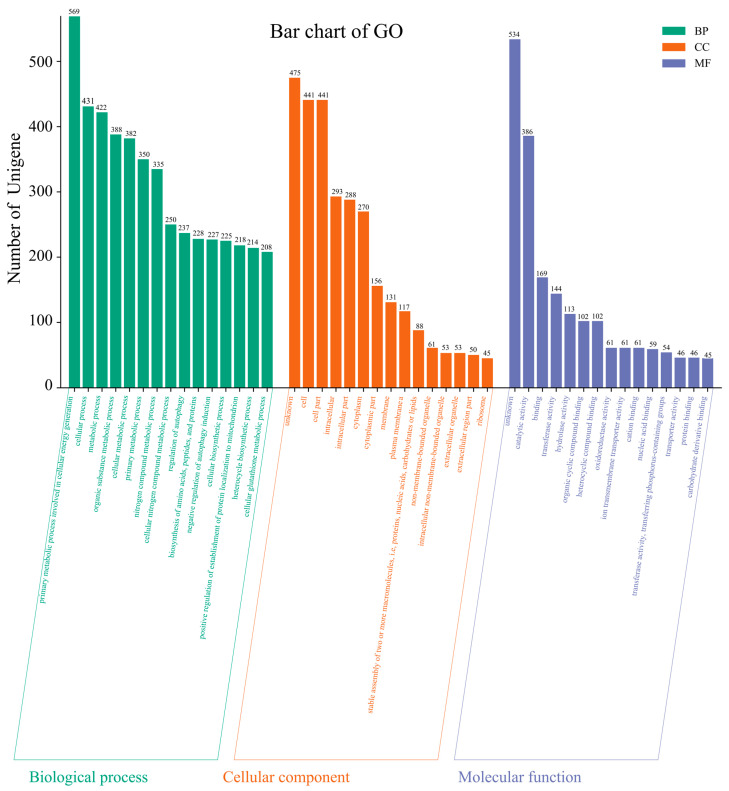
Annotations of strain ZC255 based on the Gene Ontology database.

We identified three major categories of genes in strain ZC255, which are Biological Process, Cellular Component, and Molecular Function, based on the GO database. The top fifteen subcategories are shown within each major category in the GO database in Figure 8. In the “Biological Process” category, “primary metabolic process involved in cellular energy generation” exhibited the highest value, which suggests that the metabolic processes of ZC255 have a stable energy source. Within the category “Cellular Component”, we found a relatively high proportion for “membrane”. This observation implies that strain ZC255 may have a significant number of membrane components that contribute to producing biofilms under copper stress. Moreover, we found some categories annotated as “ion transmembrane transporter activity” and “cation binding” in the category “Molecular Function”, providing evidence of the bioaccumulation capacity of strain ZC255.

**Figure 9:**
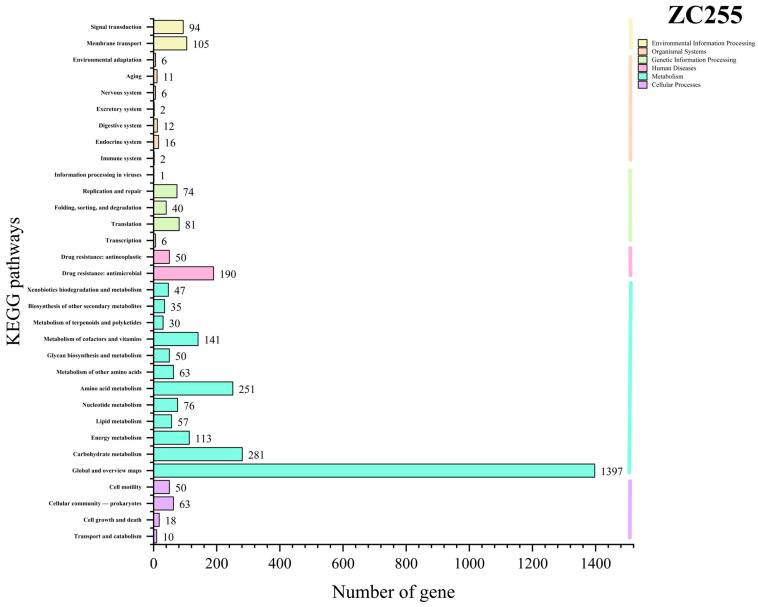
The pathway classification statistics of functional genes of strain ZC255 based on the Kyoto Encyclopedia of Genes and Genomes database.

We carried out KEGG function annotation analysis by comparing the genome of strain ZC255 with the KEGG database (Figure 9). A total of 2160 genes were annotated. According to the annotation results, strain ZC255 revealed a substantial number of genes associated with carbohydrate metabolism, thereby facilitating stable energy metabolism [66]. Simultaneously, we found two pathways annotated as “Mineral absorption”, twenty pathways annotated as “Drug resistance: antimicrobial”, and five pathways annotated as “Drug resistance: antineoplastic”, providing the basis for the drug and heavy metal resistance of the strain. In addition, twenty-six pathways were annotated as “Biofilm formation”. Biofilm formation is a defensive strategy employed by bacteria to enhance their resilience in adverse environments, thereby increasing their probability of survival [67].

As is widely acknowledged, biosorption and bioaccumulation have great potential to replace traditional methods for removing heavy metal pollution [68]. Through genomic annotation analysis, we found that strain ZC255 possesses a substantial number of functional genes related to copper accumulation, indicating its considerable potential for applications in bioremediation. The simultaneous presence of biosorption and bioaccumulation mechanisms within strain ZC255 provides compelling evidence of its latent capacity to serve as an efficient biosorbent for heavy metals. At the same time, we discovered some genes related to stress resistance conditions, providing a theoretical basis for the stress resistance of strain ZC255.

## 4. Conclusions

The experimental investigation systematically evaluated critical operational parameters, including pH, temperature, and inoculation amount, regarding the removal of Cu(II). The maximum removal capacity was 253.4 mg/g biomass under optimum conditions (pH 7.0, 28 °C, and 2% inoculation amount). Meanwhile, kinetic analysis confirmed that adsorption follows pseudo-second-order kinetics, indicating chemisorption as the dominant mechanism. Equilibrium isotherm studies revealed that adsorption behavior aligns with both the Langmuir (monolayer adsorption) and the Freundlich (heterogeneous surface) models, suggesting complex interactions between Cu(II) and bacterial surfaces. Scanning electron microscopy (SEM) revealed significant surface deformation, cell aggregation, and floc formation after Cu(II) exposure, and Fourier transform infrared spectroscopy (FTIR) showed that specific functional groups contributed to adsorption. Genomic analysis and annotation of *Rossellomorea* sp. ZC255 also identified numerous functional genes associated with the removal of and resistance to heavy metals and antibiotics. Pathways related to biofilm formation and membrane transport support extracellular and intracellular detoxification strategies. These findings position *Rossellomorea* sp. ZC255 as a robust, genomically equipped biosorbent useful for copper bioremediation. Its efficiency under mild conditions (pH 7.0 and 28 °C), coupled with multi-mechanistic adsorption capabilities, offers significant advantages for eco-friendly wastewater treatment applications.

## Data Availability

The original contributions presented in this study are included in this article. Further inquiries can be directed to the corresponding author.

## Supplementary Materials

The following supporting information can be downloaded at https://www.mdpi.com/article/10.3390/microorganisms13081839/s1. Table S1: First-order and second-order kinetics parameters were obtained for the biosorption of Cu(II) by strain ZC255. Table S2: Langmuir and Freundlich isotherm parameters obtained for the biosorption of Cu(II) by strain ZC255. Table S3: All the observed bands and their assignments from Fourier transform infrared spectroscopy (FTIR) analysis. Table S4: Functional genes associated with antibiotics.
