# Supplementary material for: Efficient Copper Biosorption by Rossellomorea sp. ZC255: Strain Characterization, Kinetic–Equilibrium Analysis, and Genomic Perspectives
Source: Microorganisms. 2025 Aug 7;13(8):1839. doi: 10.3390/microorganisms13081839 (PMC12388574; doi:10.3390/microorganisms13081839)
Supplement: Supplementary file 1 [file microorganisms-13-01839-s001.zip › microorganisms-3734865-supplementary.pdf]

Efficient Copper Biosorption by *Rossellomorea* sp. ZC255: Strain Characterization, Kinetic–Equilibrium Analysis, and Genomic Perspectives

Hao-Tong Han [a], Han-Sheng Zhu [a], Jin-Tao Zhang [a], Xin-Yun Tan [a], Yan-Xin Wu [a], Chang Liu [a], Xin-Yu Liu [a], Meng-Qi Ye* [a, b, c]

[a] Marine College, Shandong University, Weihai, Shandong, 264209, PR China.

[b] Shenzhen Research Institute, Shandong University, Shenzhen 518057, China

[c] Weihai Research Institute of Industrial Technology of Shandong University, Weihai, 264209, PR China

yemengqi@sdu.edu.cn（M.-Q.-Y）

Table S1

First–order and second–order kinetics parameters were obtained for the biosorption of Cu(II) by the strain ZC255.

	first-order kinetics			second–order kinetics		
Cu^{2+}	K_1	Q_e(mg/g)	R^2	K_2	Q_e(mg/g)	R^2
ZC255	0.0524	592.4	0.915	0.0001	598.9	0.997

Table S2

Langmuir and Freundlich isotherm parameters obtained for the biosorption of Cu(II) by the strain ZC255.

	Langmuir isotherm			Freundlich isotherm		
Cu^{2+}	K_L	q_{max}(mg/g)	R^2	K_F	n	R^2
ZC255	0.0016	4783.09	0.976	9.9998	1.099	0.972

Table S3

All the observed bands and their assignments from Fourier transform infrared spectroscopy (FTIR) analysis.

Observed peaks		Functional groups
Control	Cu^{2+} treated	
3400.89	3303.22	O-H
		N-H
-	2961.57	C-H
1659.98	1659.10	C=O
-	1544.18	N-H
		C≡N
1423.81	1404.19	C-H
-	1243.64	-COOH
1112.51	1077.97	C≡N
612.44	620.66	$-NH_2$
		$-NO_2$

Table S4

Functional genes associated with antibiotics.

COG ID	Derscription
COG 0346	Glyoxalase/Bleomycin resistance protein/Dioxygenase superfamily
COG 2076	Small Multidrug Resistance protein
COG 1846	helix_turn_helix multiple antibiotic resistance protein
COG 2720	vancomycin resistance protein
COG 0697	Putative multidrug resistance efflux transporter
COG 3570	Aminoglycoside/hydroxyurea antibiotic resistance kinase
COG 1357	Quinolone resistance protein
COG 1968	Catalyzes the dephosphorylation of undecaprenyl diphosphate (UPP). Confers resistance to bacitracin
